# Draft Genome Sequence of Cronobacter sakazakii Cr268, Isolated from Infant Powder Formula

**DOI:** 10.1128/MRA.01067-19

**Published:** 2019-11-14

**Authors:** Gal Zizelski Valenci, Mor Rubinstein, Reuven Afriat, Shira Rosencwaig, Zeev Dveyrin, Efrat Rorman, Israel Nissan

**Affiliations:** a Ministry of Health, National Public Health Laboratory, Tel Aviv, Israel; Georgia Institute of Technology

## Abstract

Cronobacter sakazakii is an emerging pathogen that causes meningitis, bacteremia, sepsis, and necrotizing enterocolitis in premature infants. Strain Cr268 was isolated from imported powdered infant formula in 2009 during routine microbial examination according to ISO-22964 (“Microbiology of the food chain—horizontal method for the detection of *Cronobacter* spp.”). Isolate Cr268 was confirmed to be C. sakazakii by matrix-assisted laser desorption ionization–time of flight mass spectrometry (MALDI-TOF MS) and standard biochemical analysis. Here, we announce its genome, which represents a new member in the C. sakazakii group.

## Abstract (precis)

Cronobacter sakazakii is an emerging pathogen that causes meningitis, bacteremia, sepsis, and necrotizing enterocolitis in premature infants. Strain Cr268 was isolated from imported powdered infant formula in 2009 during routine microbial examination according to ISO-22964 (“Microbiology of the food chain—horizontal method for the detection of *Cronobacter* spp.”).

## ANNOUNCEMENT

Cronobacter sakazakii, formerly named Enterobacter sakazakii (1), is a Gram-negative opportunistic pathogen that can be life-threatening, especially when it contaminates powdered milk formula for infants (2). It causes infections in immunocompromised individuals of all ages, particularly in infants and the elderly. Symptoms of infection include meningitis, bacteremia, sepsis, and necrotizing enterocolitis. Mortality rates are high and may reach 80% (3–7). In order to expand our knowledge about this group of pathogens, multiple confirmed C. sakazakii samples were sequenced and analyzed. In this work, we present the genome of isolate Cr268.

The isolate was revived from a frozen stock on Trypticase soy agar (TSA) at 37°C for 24 h. Using an inoculating loop, two typical colonies were suspended in 400 μl of saline and washed twice before resuspension in 200 μl of saline. Genomic DNA of C. sakazakii Cr268 was purified using a MagNA Pure Compact instrument (Roche) following the manufacturer’s instructions. DNA libraries were prepared using a Nextera DNA Flex library preparation kit following the manufacturer’s instructions. Sequencing was performed on an Illumina MiSeq platform using a 250-bp paired-end read v2 kit. All bioinformatics analyses were performed using the PATRIC v3.5.36 platform (8) with default parameters unless otherwise noted. FastQC analysis (9) confirmed that the fastq files were of good quality. The total number of reads was 1,352,469. We used the Comprehensive Genome Analysis Service, including *de novo* assembly by SPAdes (10), for analysis of this isolate. Remapping the reads to the *de novo* assembly resulted in a median base coverage of 68× and a mean base coverage of 68.8×.

The assembly produced 43 contigs with contig *L*_50_ and *N*_50_ values of 4 and 544,501 bp, respectively. The draft genome of C. sakazakii Cr268 revealed 4,477,217 bp with an average G+C content of 56.87%. Genome annotation was created with the Rapid Annotations using Subsystems Technologies tool kit (RASTtk) (11). A total of 4,338 protein-coding sequences (CDS) were predicted, as well as 77 tRNA gene sequences, 11 rRNA (rRNA) genes, a nonfunctional CRISPR system that contains only the *cas3* gene, and 2 CRISPR direct repeat (DR) arrays with 22 spacers. The genome contains genes annotated as encoding efflux pumps such as AcrAB-TolC and AcrAD-TolC and antibiotic resistance genes such as *marA* and *marB*. The multilocus sequence type (MLST) for strain Cr268 is 458 (ST458). As of October 2019, this ST was represented by only 3 isolates in the PubMLST database (https://pubmlst.org/).

The Similar Genome Finder Service, using the Mash/MinHash algorithm, identified C. sakazakii strain MOD1_KW9 (GenBank accession number NITF00000000) as the closest homolog to Cr268. We used the Average Nucleotide Identity (ANI) calculator (12) to estimate the average nucleotide identity to strain MOD1_KW9, as well as the closest high-quality C. sakazakii BAA-894 representative genome at the National Center for Biotechnology Information (NCBI; GenBank accession number NC_009778). The results of the two-way ANI calculations were 98.06% and 97.92%, respectively.

In summary, these finding indicate that strain Cr268 belongs to the C. sakazakii species.

### Data availability.

This whole-genome shotgun project has been deposited at DDBJ/ENA/GenBank under the accession number VJZG00000000. The version described in this paper is the first version, VJZG01000000. The Sequence Read Archive (SRA) submission is available under accession number SRR10023939.
